# Bilateral rectus sheath haematoma complicating dengue virus infection in a patient on warfarin for mechanical aortic valve replacement: a case report

**DOI:** 10.1186/s13104-016-2330-x

**Published:** 2017-01-07

**Authors:** Chamith Thushanga Rosa, Mitrakrishnan Rayno Navinan, Sincy Samarawickrama, Himam Hamza, Maheshika Gunarathne, Arulprashanth Arulanantham, Neeha Subba, Udari Samarasiri, Thushara Mathias, Aruna Kulatunga

**Affiliations:** 1 Internal Medicine, National Hospital of Sri Lanka, Colombo, Sri Lanka; 2 Institute of Cardiology, National Hospital of Sri Lanka, Colombo, Sri Lanka

**Keywords:** Dengue viral fever, Bleeding, Warfarin, Anticoagulation, Mechanical valves, Case report, Rectus sheath, Haematoma

## Abstract

**Background:**

The management of Dengue virus infection can be challenging. Varied presentations and numerous complications intrinsic to dengue by itself increase the complexity of treatment and potential mortality. When burdened with the presence of additional comorbidities and the need to continue compulsory medications, clear stepwise definitive guidance is lacking and patients tend to have more complex complications and outcomes calling to question the clinical decisions that may have been taken. The use and continuation of warfarin in dengue virus infection is one such example.

**Case presentation:**

We report a 65 year old South Asian female who presented with dengue fever. She had a history bronchial asthma, a prior abdominal surgery, and was on warfarin and maintained a therapeutically appropriate internationalized normalized ratio for a mechanical aortic valve replacement. Though preemptive decision to stop warfarin was taken with decreasing platelet counts, her clinical course was complicated with the development of bilateral rectus sheath haematoma’s requiring resuscitation with blood transfusions.

**Conclusion:**

Though management of dengue viral fever has seen drastic evolution with recent updated guidance, clinical scenarios seen in the course of the illness still pose challenges to the managing physician. The need to continue obligatory anticoagulation which may seem counterintuitive during a complex disease such as dengue virus infection must be considered after understanding the potential risks versus that of its benefits. Though case by case decisions maybe warranted, a clear protocol would be very helpful in making clinical decisions, as the correct preemptive decision may potentially avert catastrophic and unpredictable bleeding events.

## Background

Dengue viral fever has taken pandemic proportions as nearly half of the world’s populace is at risk [1], and rapid global urbanization ensures the further increased spread and scourge of this potentially deadly mosquito borne viral infection. The advent of a live attenuated tetravalent vaccine gives hope for the future with proven efficacy against virologically confirmed dengue [2]. But it may still be awhile before its impact is felt. Adoption has been slow with Philippines taking the initiative to introduce dengue vaccine into its public health initiative in April 2016, though amidst controversy. Dengue viral fever is renowned for its propensity to cause capillary leakage resulting in dengue haemorrhagic fever with resultant morbidity and mortality. The rarer variants of presentations are being recognized and categorized separately as dengue expanded syndrome [3]. Bleeding, however is a well-known complication of dengue and has been recognized in its varied possible phases of the disease including in dengue fever by itself in the absence of capillary leakage, during dengue haemorrhagic fever and dengue shock [4]. Though cautious fluid balance and replacement is the cornerstone of management in minimizing morbidity and mortality [1], the lack of consistency in the pattern of presentation and progression of the disease can still pose a challenge in management, even in normal circumstances for the skilled physician. When complicated with single organ involvement, bleeding or compounded with the burden of managing additional co-morbidities requiring complex simultaneous management, takes an already challenging situation into unknown territory, with little in the way of evidence based guidance. The use or continuation of anticoagulation in mandatory situations is one such example and the lack of clear guidance with regard to anticoagulation in dengue management protocols and guidelines have been noted [5]. In this case vignette we present and discuss a patient who required lifelong anticoagulation due to mechanical aortic valve replacement who presented with dengue fever and suffered complicated bleeding which posed significant challenges in regard to clinical decisions and management.

## Case presentation

A 65 year old South-Asian female presented to the medical ward with an acute febrile illness with characteristic prodromal symptoms on day 1 of her illness. Being an endemic country to dengue viral infection, clinical suspicion made us prioritize our investigations and a positive dengue NS1 antigen confirmed dengue viral infection. She had a significant past history of symptomatic aortic stenosis which necessitated surgery and had undergone mechanical aortic valve replacement in 1999. She was on oral warfarin and had maintained a therapeutically appropriate internationalized ratio (INR) while on 5 mg with no prior episodes of significant bleeding. She additionally gave a history of hypertension with dyslipidemia and was also an asthmatic on medical management with good compliance. She also had a notable obstetric past history which had required a classical caesarean section.

Preliminary examination revealed a normotensive patient with a blood pressure of 130 mmHg systole and 80 mmHg diastole and had a regular pulse of 92 beats per minute. Cardiac auscultation revealed a mechanical click coinciding with 2nd heart sound and an ejection systolic murmur favoring a functional mechanical aortic valve in the absence of any other findings favoring decompensation. Though respiratory examination revealed scattered bilateral rhonchi, the patient appeared stable with a respiratory rate of 16 breaths per minute. Abdominal examination revealed a midline scar keeping in with the history of a classical caesarean section. Neurological examination was unrevealing. A decision was taken to continue her routine metered dose inhalers of salmeterol and fluticasone 250/50 micrograms twice daily. Additionally she was also nebulized 8 hourly with oxygen driven salbutamol. Though she had been on oral losartan 50 mg bd for her hypertension and rosuvastatin 10 mg nocte for her dyslipidemia a clinical decision was taken to discontinue both. However in consideration of her mechanical aortic valve, we took the clinical decision to continue her warfarin without dose reduction at 5 mg vesper.

On admission, which was day 1 of her illness, whole blood analysis revealed a hemoglobin of 9.3 g/dL (11–15) with a low total white cell count of 3.89 × 10^9^/L (4–11) and a platelet count of 113 × 10^9^/L (150–450). Her INR was 2.7 (<1 normal and target range of 2–3 in her situation). Warfarin was cautiously continued while judiciously following her drop in platelet counts with frequent whole blood analysis. Her liver functions showed a mild but acceptable derangement of transaminases with an aspartate transaminase of 144 (<35 IU) and alanine transaminase of 62 (<40 IU) with a normal reference range for activated thromboplastin time. On day 4 of her illness when her platelet count dropped to below 50 × 10^9^/L (150–450), warfarin was stopped and a clinical decision was taken to change over to intravenous unfractionated heparin (at 1000 IU/h) as the anticoagulant of choice to ensure continued anticoagulation for her mechanical heart valve. She was closely monitored with activated partial thromboplastin time (APTT) at six hourly intervals. However, a rapid and unexpected prolongation of APTT to >200 s (<40 s) prompted us to reconsider continuation of heparin and decision was taken to transiently withhold heparin. During this period she was neither symptomatic nor had clinically evident signs of bleeding or its manifestations. She was continuously monitored and on day 6 her platelets continued to drop to its lowest nadir of 27 × 10^9^/L (150–450). Her deranged APTT had normalized by this point and were within normal reference parameters.

**Fig. 1:**
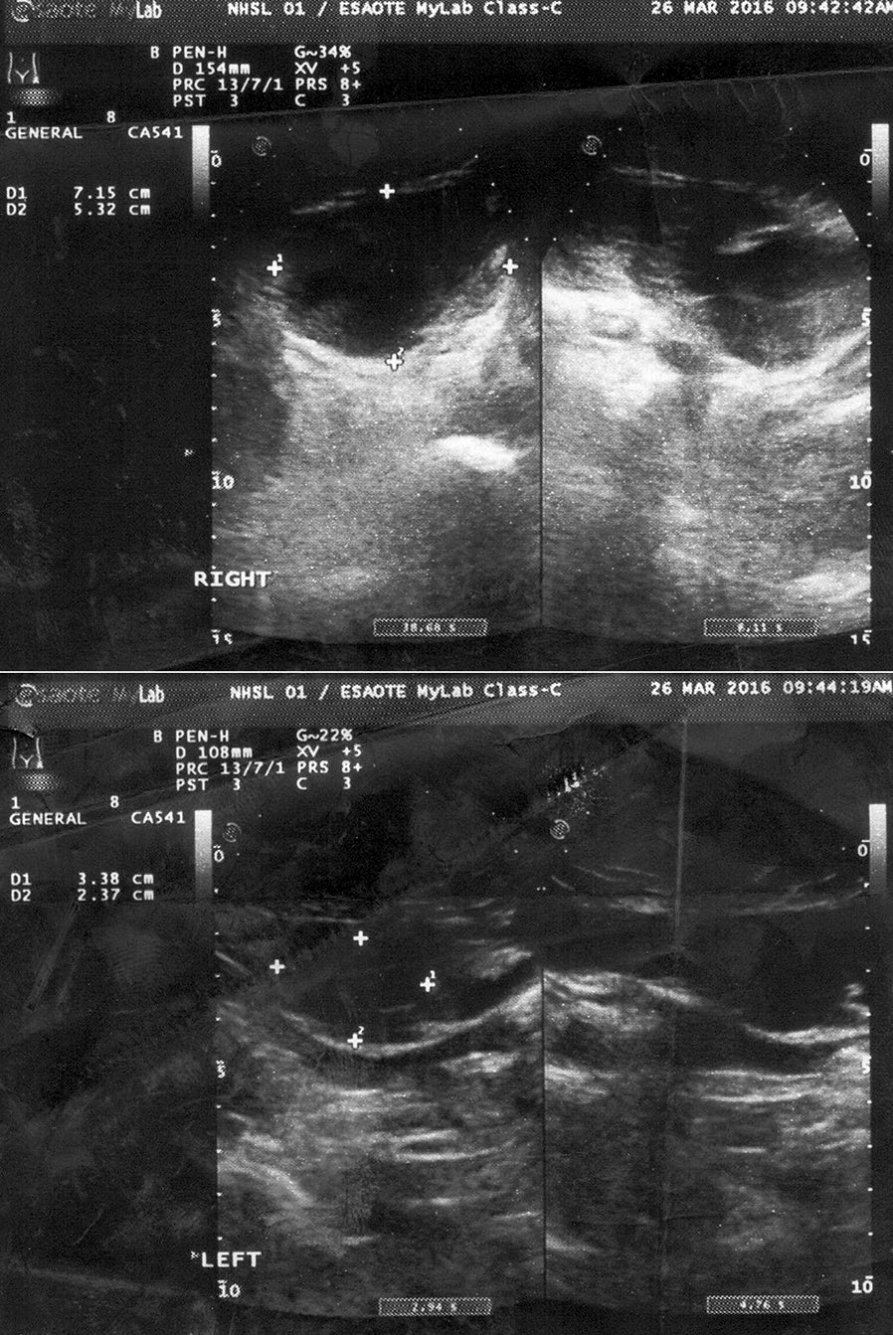
Image depicts the ultrasound findings which reveal a right sided hypo-echoic lesion (*upper image*) within the rectus sheath measuring 71 mm by 53 mm (depicted by cross hairs) and similar lesion within the left side rectus (*lower image*) sheath measuring 33 mm by 23 mm (depicted by cross hairs)

On day 7 of illness with an already rising platelet count of 50 × 10^9^/L (150–450), and normal coagulation parameters (an INR of 1.1 and APTT of 35 s) she complained of a continuous lower abdominal pain. However clinical examination was unrevealing. While still being monitored with frequent whole blood analysis an observation was made of a drop in both haemoglobin and haematocrit from 9.3 g/dL (11–15) to 7.9 g/dL (11–15) and from 33 (35–45) to 27 (35–45) respectively, which also coincided with a notable drop in her urine output (<0.5 cc/kg/h) which had maintained till then. Despite a tachycardia of 110 beats per minute her blood pressure remained within acceptable parameters, suggesting compensated shock. A possible intra-abdominal bleed was suspected. Ultrasonographic examination revealed a right sided rectus sheath haematoma measuring 13 mm by 15 mm. There were however no features to favor dengue haemorrhagic fever as features of fluid leakage were absent ultrasonographically. Failure of the parameters to correct, prompted us to do a follow up ultrasound abdomen which confirmed the extension of the haematoma on the right to 71 mm by 53 mm with a new haematoma forming on the contralateral left side as well, measuring 33 mm by 23 mm (Fig. 1). No further bleeding sites were identified. Additional occult bleeding was suspected and based on haematological and haemodynamic parameters, she was initially volume resuscitated with normal saline and to err on the side of caution she was also transfused with 3 units of packed red cells to achieve stabilization and counteract blood loss. Post transfusion haemoglobin was 10.1 g/dL (11–15) and haematocrit was 34 (35–45). A decision was taken not to transfuse fresh frozen plasma or platelets as tested coagulation parameters (APTT & INR) were normal, and platelet counts were 50 × 10^9^/L (150–450) and rising. With defervescence of temperature and rising platelets, her haematological parameters stabilized and intensive monitoring was continued. After 72 h of clinical stability she was started on oral anticoagulation and bridged with low molecular heparin until the INR reached acceptable therapeutic range. The patient thereafter made an uncomplicated recovery.

## Conclusions

Dengue viral fever has a myriad of presentations and complications. Bleeding is an acknowledged and notorious complication of dengue. Through complex pathophysiological mechanisms dengue fever can impact the risk and cause bleeding in many ways. Hypothesis have been put forward questioning the role of hematological disturbance through thrombocytopenia and platelet dysfunction, to low fibrinogen and factors involved in the coagulation cascade resulting in prolonged APTT. Hepatic dysfunction and coagulopathy worsened by disseminated intravascular coagulation and even vasculopathy are thought to possibly contribute towards bleeding, aided by the immunological disturbance seen in dengue viral fever [6–8]. When coupled together with use of either anti-platelet’s or anticoagulation the theoretical risk of bleeding is exponentially worse.

Warfarin is one of the most commonly used anticoagulants due to affordability, availability and proven efficacy. The factors that contribute towards bleeding are many and the risk differs on individual basis. But overall the risk of bleeding is greatest when initiating warfarin, or if the dose was supra-therapeutic, if there were drug interactions and when complicated with an ongoing illness. In fact a dose reduction is advised during inter-current illness. Advancing age is another confounding factor and there is still uncertainty whether it acts as an independent factor. Additionally when burdened with chronic diseases such as hypertension and renal insufficiency, the likelihood of bleeding is even greater when using warfarin [9, 10].

Bleeding can occur in any part of the body when using anticoagulation. Rectus sheath haematoma is a rare yet observed complication of anticoagulation [11]. Bilateral rectus sheath haematoma is an even rarer occurrence with few documented cases in literature [12–14]. Rectus sheath haematoma is commonly seen in women more than men and specially in their 6th to 7th decades of life [15]. The risk of developing rectus sheath haematoma is greater when having a background history of trauma, straining, coughing, pregnancy and abdominal surgery [16]. Rarely, rectus sheath haematomas have also been documented complicating dengue viral fever [14, 17]. Though conservative management is adequate in most circumstances with the cessation of anticoagulation, when complicated with haemodynamic instability supportive care coupled with blood transfusion may become necessary [11, 15]. Thus having awareness and maintaining a high index of suspicion in appropriate clinical circumstances is paramount for the early and accurate diagnosis of rectus sheath haematoma. This will result in the opportunity for quick institution of appropriate treatment measures, with improved outcome [18].

There are obligatory conditions where the use of anticoagulation is essential and needs to be continued as the benefit outweighs the potential risks. When bleeding occurs the choice of discontinuing or reducing oral anticoagulation depends on whether the episode was a major or minor event [19]. When compounded with an ongoing dengue viral infection the decision to continue anticoagulation challenges the physician to reassess its need. However a definitive clinical event such as an established bleed while having dengue viral fever may simplify the difficult clinical decision of stopping anticoagulation out of necessity.

In the absence of either obvious or occult bleeding, the clinician may choose to cease anticoagulation in the presence of dropping platelet counts to err on the side of caution. The point at which this has to be done should be decided on a case by case basis. The lowest threshold of absolute platelet count considered safe has varied, but usually lies between the range of 50 × 10^9^/L (150–450) to 100 × 10^9^/L (150–450) [5, 20]. The safe limit suggested to reinitiate anticoagulation is when platelet counts rise above 50 × 10^9^/L (150–450) [5, 20].

The possible justifications to argue in favor of this course of action are twofold. Foremost in the presence of life threatening bleeds, anticoagulation should be discontinued even in obligatory circumstances. In such situations anticoagulation has been discontinued as long as by a week, and sub-therapeutic levels have been maintained even further successfully with minimal thromboembolic risk [5]. Additionally the annual risk of thrombosis with mechanical valves even without anticoagulation is only 20% [8] and thus may justify the decision. The second consideration being is the persistence of anticoagulation to a certain degree even following cessation. In average it takes 2–3 days for the INR to drop below 2, and nearly 4–6 days for it to normalize [21]. The single most important factor that determines this is the initial baseline INR level [22]. Based on this, even a higher level platelet count could be justified when stopping warfarin if the INR is inappropriately elevated at the outset. Thus a level of anticoagulation being present even following cessation may offer some degree of protection and theoretically grants a few days of bargaining time. That being said, the drop in platelet counts in dengue fever is also quite variable, and hard to predict as significant drop occurs in 3rd to 4th day of fever and the counts keep decreasing to an average of 7 days [23].

Despite all of this there is still disagreement as to if bleeding is associated with thrombocytopenia, even when very severe [7, 23]. The problem is further compounded by the fact that bleeding in dengue viral fever is probably multifactorial as mentioned above, and choosing only absolute platelet counts, whatever the count value as a sole criterion to stop and restart anticoagulation may in fact be an arbitrary decision, as it happens to be the easiest one to monitor.

The patient along with their primary need of anticoagulation and their associated comorbidities should be taken into consideration when making the clinical decision to cease anticoagulation. To achieve this, patients can be risk stratified. A higher risk is present when the need is following mechanical valve replacement, and more so if the replacement involves multiple valves. A prior history of thromboembolic episodes and having added comorbidities increases one risk (e.g. diabetes, hypertension or prior stroke), and places an individual into a high risk category as well. In such situations withholding warfarin and substituting heparin when INR becomes sub-therapeutic and omitting all forms of anticoagulation when platelets drop below 50 × 10^9^/L (150–450) or even earlier in advanced forms of presentation of dengue spectrum with events of either bleeding or shock have been suggested. Alternatively when having low risk factors e.g. chronic atrial fibrillation with minimal risk factors, consider omitting warfarin to err on the side of caution by as much as one week without having stringent criteria is preferred [8, 24].

Our patient had all the classic and added risk factors needed to develop this rare yet potentially lethal complication, including cough secondary to bronchial asthma, prior abdominal surgery and distorted anatomy and developing dengue viral fever while being on warfarin. The fact that we preemptively stopped anticoagulation (both warfarin and unfractionated heparin) due to her high risk status, and her coagulation parameters being normal at the point in time of her haemorrhage points towards another cause, other than the anticoagulation as the cause for her to have bled. Thrombocytopenia though attributable to dengue viral fever could also have rarely resulted from heparin, in the form of heparin induced thrombocytopenia (HIT), type 1. However, HIT is predominantly pro-thrombotic and thus the propensity to bleed in the presence of thrombocytopenia favors dengue more than that of HIT. Though her other risk factors may have played a role in contributing towards the resultant bleed, one should strongly consider the role dengue viral fever had to play. Considering the event in question occurred during convalescence as commonly observed in other case reports of similar nature with significant bleeding [14], we can possibly assume that dengue viral fever through its complex mechanism may have been the primary cause for her to have bled.

The decision to continue or stop anticoagulation may never be an easy choice to make in dengue viral infection unless complicated with a bleeding event. In the absence of which a patient based risk stratification should be done considering all relevant information including the need for anticoagulation and any associated comorbidities that may inadvertently facilitate the risk of bleeding. Making the correct decision to stop anticoagulation in a timely manner may potentially help prevent catastrophic bleeding events considering the highly unpredictable nature of dengue viral infection and its propensity to cause bleeding in different phases of illness through complex mechanisms.

The clinical management of patients with dengue viral fever on mandatory anticoagulation is difficult, as the decision to continue or cease warfarin compels the physician to weigh the risks intrinsic to dengue fever versus that of lack of anticoagulation. There is limited guidance, and even the available guidance is based on case reports and extrapolations from similar clinical scenarios. The unpredictable course of dengue viral fever and the lack of understanding of the underlying pathophysiological process that predisposes to bleeding in dengue viral fever makes even the well thought out decision to stop anticoagulation in retrospect to appear arbitrary. However based on risk factors, making the correct decision in a timely manner to withhold anticoagulation can potentially avert catastrophic clinical events. Thus it should be stressed, if a decision is taken to cease anticoagulation in dengue viral fever it should be based on a case by case basis considering all relevant risk factors and the evolving clinical picture. Though lacking, the presence of a clear protocol and guidance would greatly aid clinicians when faced with making such difficult decisions.

## Abbreviations

INR: international normalized ratio
APTT: activated partial thromboplastin time
HIT: heparin induced thrombocytopenia
